# Stable Sheaves on a Smooth Quadric Surface with Linear Hilbert Bipolynomials

**DOI:** 10.1155/2014/346126

**Published:** 2014-02-11

**Authors:** Edoardo Ballico, Sukmoon Huh

**Affiliations:** ^1^Università di Trento, 38123 Povo, Italy; ^2^Sungkyunkwan University, Suwon 440-746, Republic of Korea

## Abstract

We investigate the moduli spaces of stable sheaves on a smooth quadric surface with linear Hilbert bipolynomial in some special cases and describe their geometry in terms of the locally free resolution of the sheaves.

## 1. Introduction

Throughout the paper, our base field is *ℂ*, the field of complex numbers.

By the work of Simpson [1], we can consider the moduli space of semistable sheaves on a smooth projective variety *X* with a fixed Hilbert polynomial, which is itself a projective variety, and the moduli space has been studied quite intensively in the last decade for the case with linear Hilbert polynomial over projective spaces [2–5]. Our interest is on the moduli space over a smooth quadric surface.

Let *Q* be a smooth quadric surface in *ℙ* ^3^ and let **M**_*Q*_(*μ*, *χ*) be the moduli space of semistable sheaves on *Q* with linear Hilbert polynomial *χ*(*m*) = *μm* + *χ* with respect to the ample line bundle *𝒪*_*Q*_(1,1). Unlike the case of projective spaces, this moduli space is not irreducible in general. Indeed, for a purely 1-dimensional sheaf *ℱ* on *Q*, we can define a linear Hilbert bipolynomial *χ*_*ℱ*_(*x*, *y*) such that

(1) $$\begin{array}{c} \chi \left(ℱ\left(x,y\right)\right)=\chi_{ℱ}\left(x,y\right) \end{array}$$

for all (*x*, *y*) ∈ *ℤ* ^⊕2^. Then we can consider, due to [6], the moduli space **M**(*m*, *n*, *t*) of semistable sheaves on *Q* with linear Hilbert bipolynomial *χ*(*x*, *y*) = *mx* + *ny* + *t*. The moduli space is a projective variety with a Zariski open subset **M**°(*m*, *n*, *t*) consisting of stable ones, with dimension 2*mn* + 1 and the open set is nonempty if one of *m* or *n* is nonzero (see Proposition 7).

By its definition we have a natural decomposition

(2) $$\begin{array}{c} M_{Q}\left(m+n,t\right)=\underset{0\leq a\leq m+n}{∐}M\left(a,m+n-a,t\right). \end{array}$$

Thus, the moduli **M**(*m*, *n*, *t*) is an irreducible component of Simpson's moduli space because the bidegree function is locally constant.

If *ℱ* is a stable sheaf in **M**(*m*, *n*, *t*), then its schematic support *C*_*ℱ*_ is a curve of bidegree (*n*, *m*) on *Q* and so a general sheaf is a line bundle over a smooth subcurve. Thus, the moduli space can be considered as an analogue of the universal line bundle *𝒫ic*_(*n*,*m*)_^*d*^ of some fixed degree *d* over the family of the bidegree (*n*, *m*)-curves in *Q*.

Now, some simple observations lead us to consider only **M**(*m*, *n*, *t*) with 0 ≤ *t* ≤ gcd(*m*, *n*) due to proper twists. For small *m* or *n*, the moduli space is very simple. Indeed, **M**(*n*, 0, *t*) is isomorphic to *ℙ* ^*n*^ if *t* = *n* and is empty otherwise. If *m* or *n* is equal to 1, say *m* = 1, then it is isomorphic to *ℙ* ^2*n*+1^. These descriptions are quite simple from the definition of stability condition and so the first nontrivial case happens when (*m*, *n*) = (2,2). The main result of this paper is to describe the moduli spaces **M**(2,2, *t*) with *t* = 1,2.

**Theorem 1**

For **M**_*t*_ = **M**(2,2, *t*), one obtains the following:

1. **M**_1_ is isomorphic to *𝒫ic*_(2,2)_^1^ and it is rational;
2. **M**_2_ is birational to *𝒫ic*_(2,2)_^2^ and it is unirational with degree 4.

In fact, we explicitly describe the sheaves in each moduli space in terms of their locally free resolution. Indeed, a sheaf *ℱ* is in **M**_1_ if and only if it admits a resolution

(3) 0⟶𝒪Q(−2,−1)⊕𝒪Q(−1,−2)⟶𝒪Q⊕𝒪Q(−1,−1)⟶ℱ⟶0,

where the degeneracy locus of the first map is the support of *ℱ*. It enables us to identify **M**_1_ with *𝒫ic*_(2,2)_^1^ and show its rationality.

For **M**_2_, the situation is a bit more complicated; we can classify the sheaves in **M**_2_ up to 3 types, in terms of the short exact sequences they admit, and express the moduli as the union of 3 subschemes

(4) $$\begin{array}{c} M_{2}=𝔄\cup 𝔅\cup ℭ. \end{array}$$

In particular, we can show that every sheaf in **M**_2_ is globally generated, from which we obtain a resolution that they admit:

(5) $$\begin{array}{c} 0⟶{𝒪}_{Q}{\left(-1,-1\right)}^{⊕2}⟶{𝒪}_{Q}^{⊕2}⟶ℱ⟶0. \end{array}$$

We investigate the property of the subvarieties and the relationship between them. We also construct a map from **M**_1_ to **M**_2_, which is generically 4 to 1 and thus we obtain that **M**_2_ is unirational of degree 4. We leave the rationality question of **M**_2_ as a conjecture.

## 2. Preliminaries

Let *Q* be a smooth quadric surface isomorphic to *ℙV*_1_ × *ℙV*_2_ for 2-dimensional vector spaces *V*_1_ and *V*_2_, and then it is embedded into *ℙ* ^3^≅*ℙV* by the Segre map where *V* = *V*_1_ ⊗ *V*_2_. If we denote by *f*_1_, *f*_2_ the two projections from *Q* to each factor, then we will denote *f*_1_*𝒪_*ℙ*^1^_(*a*) ⊗ *f*_2_*𝒪_*ℙ*^1^_(*b*) simply by *𝒪*_*Q*_(*a*, *b*). We also denote *ℰ* ⊗ *𝒪*_*Q*_(*a*, *b*) by *ℰ*(*a*, *b*) for a coherent sheaf *ℰ* on *Q* and in particular the canonical sheaf *ω*_*Q*_ of *Q* is *𝒪*_*Q*_(−2, −2).

**Proposition 2**

For a purely 1-dimensional sheaf *ℱ* on *Q*, there is a bipolynomial *χ*_*ℱ*_(*x*, *y*) ∈ *ℚ*[*x*, *y*] of degree 1 such that

(6) $$\begin{array}{c} \chi \left(ℱ\left(u,v\right)\right)=\chi_{ℱ}\left(u,v\right) \end{array}$$

for all (*u*, *v*) ∈ *ℤ* ^⊕2^.

**Proof**

Let us assume that *mt* + *c* is the Hilbert polynomial of *ℱ* with respect to the ample line bundle *𝒪*_*Q*_(1,1). Let us take any *D* ∈ |*𝒪*_*Q*_(0,1)|, *T* ∈ |*𝒪*_*Q*_(1,0)|, and a smooth conic *C* ∈ |*𝒪*_*Q*_(1,1)| such that neither *D*, *T*, nor *C* is contained in the 1-dimensional reduced curve Supp(*ℱ*).

The curves *D*, *T*, and *C* induce maps *j*_*D*_ : *ℱ*(*t*, *t*) → *ℱ*(*t*, *t* + 1),  *j*_*T*_ : *ℱ*(*t*, *t*) → *ℱ*(*t* + 1, *t*), and *j*_*C*_ : *ℱ*(*t*, *t*) → *ℱ*(*t* + 1, *t* + 1). Since neither *D* nor *T* is contained in the 1-dimensional reduced curve Supp(*ℱ*), we have *j*_*D*_ ≠ 0 and *j*_*T*_ ≠ 0. Since *ℱ* is pure, we obtain that *j*_*D*_, *j*_*T*_, and *j*_*C*_ are injective. Thus, there are exact sequences

(7) 0⟶ℱ(t,t)⟶ℱ(t,t+1)⟶ℱ(t,t+1)⊗𝒪D⟶0,

(8) 0⟶ℱ(t,t)⟶ℱ(t+1,t)⟶ℱ(t+1,t)⊗𝒪T⟶0,

(9) 0⟶ℱ(t,t)⟶ℱ(t+1,t+1)⟶ℱ(t+1,t+1)⊗𝒪C⟶0.

Let us set *a* : = *h* ^0^(*ℱ*(*t*, *t* + 1) ⊗ *𝒪*_*D*_) and *b* : = *h* ^0^(*ℱ*(*t* + 1, *t*) ⊗ *𝒪*_*T*_). The sheaves *ℱ*(*t*, *t* + 1) ⊗ *𝒪*_*D*_, *ℱ*(*t* + 1, *t*) ⊗ *𝒪*_*T*_, and *ℱ*(*t* + 1, *t* + 1) ⊗ *𝒪*_*C*_ have finite supports and thus the dimensions of their cohomology *H* ^0^(*Q*, −) do not change even if we twist them by a line bundle on *Q*. From (9), we get *a* + *b* = *h* ^0^(*ℱ*(*t* + 1, *t* + 1) ⊗ *𝒪*_*C*_) = *m*.

We claim that *χ*(*ℱ*(*u*, *v*)) = *av* + *bu* + *c* for all (*u*, *v*) ∈ *ℤ* ^⊕2^. If *u* = *v*, then the claim is true. Now assume that *u* ≠ *v*, say *u* > *v*. We use *u* − *v* exact sequences like (8) with *ℱ*(*c*, 0) instead of *ℱ* with 0 ≤ *c* < *u* − *v* to get *χ*(*ℱ*(*u*, *v*)) = *χ*(*v*, *v*)+(*u* − *v*)*b*.

**Definition 3**

One defines the Hilbert bipolynomial *χ*_*ℱ*_(*x*, *y*) ∈ *ℚ*[*x*, *y*] of *ℱ* to be a linear bipolynomial such that

(10) $$\begin{array}{c} \chi_{ℱ}\left(x,y\right)=\chi \left(ℱ⊗{𝒪}_{Q}\left(x,y\right)\right). \end{array}$$

In particular, the Hilbert polynomial of *ℱ* with respect to *𝒪*_*Q*_(1,1) is defined to be *χ*_*ℱ*_(*t*) = *χ*_*ℱ*_(*t*, *t*).

We are mainly interested in the case when *χ*_*ℱ*_(*x*, *y*) is a linear function, that is, *χ*_*ℱ*_(*x*, *y*) = *mx* + *ny* + *t* for some (*m*, *n*, *t*) ∈ *ℤ* ^⊕3^.

**Definition 4**

Let *ℱ* be a pure sheaf of dimension 1 on *Q* with *χ*_*ℱ*_(*x*, *y*) = *mx* + *ny* + *t*. The *p*-slope of *ℱ* is defined to be *p*(*ℱ*) = *t*/(*m* + *n*). *ℱ* is called semistable (stable) with respect to the ample line bundle *𝒪*_*Q*_(1,1) if

(1) *ℱ* does not have any 0-dimensional torsion,
(2) for any proper subsheaf *ℱ*′, one has
  (11) $$\begin{array}{c} p\left({ℱ}^{\prime }\right)=\frac{t^{\prime }}{m^{\prime }+n^{\prime }}\leq \left(<\right)\frac{t}{m+n}=p\left(ℱ\right), \end{array}$$
 where *χ*_*ℱ*′_(*x*, *y*) = *m*′*x* + *n*′*y* + *t*′.

For every semistable 1-dimensional sheaf *ℱ* with *χ*_*ℱ*_(*x*, *y*) = *mx* + *ny* + *t*, let us define *C*_*ℱ*_ : = Supp(*ℱ*) to be its scheme-theoretic support and then we have *C*_*ℱ*_ ∈ |*𝒪*_*Q*_(*n*, *m*)|. We often use slope stability and slope semistability instead of Gieseker stability or Gieseker semistability just to simplify the notation; they should be the same because the support is 1-dimensional, and from *mt* + *χ* and *m*′*t* + *χ*′, the inequality for Hilbert and slopes *χ*/*m* the same.

**Definition 5**

Let **M**(*m*, *n*, *t*) be the moduli space of semistable sheaves on *Q* with linear Hilbert bipolynomial *χ*(*x*, *y*) = *mx* + *ny* + *t*.

We can define **M**(*m*, *n*, *t*) in a different way as a subvariety of **M**_*Q*,*ℙ*^3^_(*m* + *n*, *t*), the moduli space of semistable sheaves on *ℙ* ^3^ with linear Hilbert polynomial *χ*(*x*) = *mx* + *t*, which are *𝒪*_*Q*_-sheaves. To be precise, if *ℱ* is *𝒪*_*Q*_-sheaf, then all of its *𝒪*_*ℙ*^3^_-subsheaves are also *𝒪*_*Q*_-sheaves. It implies that the notions of *p*-stability and *μ*-stability of *ℱ* are the same and thus **M**_*Q*,*ℙ*^3^_(*m* + *n*, *t*) may be defined without using *ℙ* ^3^. Moreover, the sheaf with linear Hilbert bipolynomial *χ*(*x*, *y*) = *ax* + *by* + *c* has Hilbert polynomial *χ*(*x*) = (*a* + *b*)*x* + *c* with respect to *𝒪*_*Q*_(1,1) and thus we have a natural decomposition

(12) $$\begin{array}{c} M_{Q,{\mathbb{P}}^{3}}\left(m+n,t\right)=\underset{0\leq a\leq m+n}{∐}M\left(a,m+n-a,t\right). \end{array}$$

In particular, **M**(*m*, *n*, *t*) is a subvariety of **M**_*Q*,*ℙ*^3^_(*m* + *n*, *t*).

**Remark 6**

Let *ℱ* be any purely 1-dimensional coherent sheaf on *ℙ* ^3^ with Hilbert polynomial *mx* + *ti*. Assume that *ℱ* is not semistable and let

(13) $$\begin{array}{c} 0={ℱ}_{0}\subset {ℱ}_{1}\subset \cdots \subset {ℱ}_{k}=ℱ \end{array}$$

be the Harder-Narasimhan filtration of *ℱ* (see page 55 in [1]). If *ℱ* is an *𝒪*_*X*_-module, then each *ℱ*_*i*_ is an *𝒪*_*X*_-module because it is a subsheaf of *ℱ*. Thus the Harder-Narasimhan filtration of *ℱ* as an *𝒪*_*ℙ*^*n*^_-sheaf is the same as the one as an *𝒪*_*X*_-sheaf.

**Proposition 7**

The moduli **M**(*m*, *n*, *t*) is a projective and irreducible scheme. If *mn* > 0, then **M**°(*m*, *n*, *t*) is a Zariski dense and open subset of **M**(*m*, *n*, *t*) with dimension 2*mn* + 1.

**Proof**

The first assertion follows verbatim from the proof of Proposition 2.3 and Theorem 3.1 in [6], only when the assertion in Lemma 3.3 over *Q* holds. But it holds, using Castelnuovo-Mumford criterion with the Serre duality

(14) H1(ℰxt1(ℱ,ℱ(j,j)))≅Ext2(ℱ,ℱ(j,j))≅Hom(ℱ(j,j),ℱ(−2,−2))∨=0

for *ℱ* ∈ **M**(*m*, *n*, *t*) and *j* ≥ −1.

For the second assertion, let us consider a map

(15) $$\begin{array}{c} M\left(m^{\prime },n^{\prime },t^{\prime }\right)\times M\left(m^{\prime \prime },n^{\prime \prime },t^{\prime \prime }\right)⟶M\left(m,n,t\right) \end{array}$$

defined by sending (*ℱ*′, *ℱ*′′) to *ℱ*′ ⊕ *ℱ*′′, where *m* = *m*′ + *m*′′ and *n* = *n*′ + *n*′′. Then the dimension of the image of this map is at least 2*mn* − 2*m*′*n*′ − 2*m*′′*n*′′ − 1 and it is at least 1 if *mn* > 0. In other words, general sheaf in **M**(*m*, *n*, *t*) is stable.

For any pure sheaf *ℱ* on *Q* with Hilbert bipolynomial *χ*_*ℱ*_(*x*, *y*) = *mx* + *ny* + *t*, let us define

(16) $$\begin{array}{c} {ℱ}^{D}:=ℰxt_{Q}^{1}\left(ℱ,\omega_{Q}\right) \end{array}$$

to be the Grothendieck dual of *ℱ*. Since *ℱ* is pure, the natural map *φ*_*ℱ*_ : *ℱ* → *ℱ* ^*DD*^ is injective. Since the support of *ℱ* is 1-dimensional, *φ*_*ℱ*_ is bijective as in Remark 4 of [4]. Moreover, the support of *ℱ* ^*D*^ is also 1-dimensional and so *χ*_*ℱ*^*D*^_(*x*, *y*) is also linear. By the Serre duality, we have

(17) $$\begin{array}{c} H^{i}\left({ℱ}^{D}\left(c,d\right)\right)≅H^{i}\left({\left(ℱ\left(-c,-d\right)\right)}^{D}\right)≅H^{1-i}{\left(ℱ\left(-c,-d\right)\right)}^{\vee } \end{array}$$

for *i* ∈ {0,1} and, in particular, we have

(18) $$\begin{array}{c} \chi_{{ℱ}^{D}}\left(x,y\right)=-\chi_{ℱ}\left(-x,-y\right)=mx+ny-t. \end{array}$$

**Lemma 8**

There is an isomorphism

(19) $$\begin{array}{c} M\left(m,n,t\right)⟶M\left(m,n,-t\right) \end{array}$$

sending *ℱ* to *ℱ* ^*D*^.

Note also that *χ*_*ℱ*(*d*,*e*)_(*x*, *y*) = *mx* + *ny* + *t* + (*md* + *ne*). Since the map

(20) $$\begin{array}{c} M\left(m,n,t\right)⟶M\left(m,n,t+md+ne\right), \end{array}$$

defined by *ℱ* ↦ *ℱ*(*d*, *e*), is an isomorphism, so we may assume that 0 < *t* ≤ gcd(*m*, *n*).

**Lemma 9**

For a not necessarily integral curve *C* in |*𝒪*_*Q*_(*n*, *m*)|, the sheaf *𝒪*_*C*_ is semistable. If *C* is integral, then *𝒪*_*C*_ is stable.

**Proof**

We have the following sequence:

(21) $$\begin{array}{c} 0⟶{𝒪}_{Q}\left(-n,-m\right)⟶{𝒪}_{Q}⟶{𝒪}_{C}⟶0. \end{array}$$

In particular, we have *χ*_*𝒪*_*C*__(*x*, *y*) = *mx* + *ny* + (*m* + *n* − *mn*) and so *p*(*𝒪*_*C*_) = 1 − 1/(1/*m* + 1/*n*). If *C* is integral, then *𝒪*_*C*_ is stable since every line bundle on an integral curve is stable. In general, *𝒪*_*C*_ is semistable. Otherwise, there exists a semistable quotient sheaf *𝒪*_*C*_ → *ℱ* → 0 such that the Hilbert bipolynomial *χ*_*ℱ*_(*x*, *y*) = *m*′*x* + *n*′*y* + *t*′ satisfies *m*′ + *n*′ < *m* + *n* and *p*(*ℱ*) < *p*(*𝒪*_*C*_). By induction, we get that *𝒪*_*C*′_ with *C*′ : = *C*_*ℱ*_ is semistable and thus we have

(22) $$\begin{array}{c} p\left({𝒪}_{C^{\prime }}\right)\leq p\left(ℱ\right)<p\left({𝒪}_{C}\right). \end{array}$$

This is absurd since *p*(*𝒪*_*C*_) is a decreasing function on *m* and *n*.

Let us assume that *m* = 0, that is, Hilb_*Q*_(*ny* + *t*) with 0 < *t* ≤ *n*.

**Proposition 10**

One has

(23) $$\begin{array}{c} M\left(0,n,t\right)≅\{\begin{array}{cc} {\left({\mathbb{P}}^{1}\right)}^{\left[n\right]}≅{\mathbb{P}}^{n} & if\;\;t=n;\;\\ \emptyset & if\;\;0<t<n.\; \end{array} \end{array}$$

In fact, each point in *Hilb*_*Q*_(*ny* + *n*) corresponds to an equivalence class [*𝒪*_*L*_1__ ⊕ ⋯⊕*𝒪*_*L*_*n*__], where *L*_*i*_ is a line in |*𝒪*_*Q*_(1,0)|.

**Proof**

Let us assume that *t* = *n* and let us choose *L* ∈ |*𝒪*_*Q*_(*n*, 0)| and then it fits into

(24) $$\begin{array}{c} 0⟶{𝒪}_{Q}\left(-n,0\right)⟶{𝒪}_{Q}⟶{𝒪}_{L}⟶0. \end{array}$$

Thus we have

(25) χ𝒪L(x,y)=χ𝒪Q(x,y)−χ𝒪Q(−n,0)(x,y)=(x+1)(y+1)−(x−n+1)(y+1)=ny+n.

Clearly, *𝒪*_*L*_ is stable. For a line *L* ∈ |*𝒪*_*Q*_(1,0)|, we have

(26) $$\begin{array}{c} \chi_{{𝒪}_{2L}}\left(x,y\right)=\chi_{{𝒪}_{L}⊕{𝒪}_{L}}\left(x,y\right)=2y+2. \end{array}$$

From the sequence for *L*, we have

(27) 0⟶Hom(𝒪L,𝒪L)⟶Hom(𝒪Q,𝒪L)⟶fHom(𝒪Q(−1,0),𝒪L)⟶Ext⁡1(𝒪L,𝒪L)⟶0

and the map *f* is a zero map. Thus, there exists a nontrivial extension of *𝒪*_*L*_ by *𝒪*_*L*_ and it is *𝒪*_2*L*_. In particular, *𝒪*_*L*_^⊕2^ and *𝒪*_2*L*_ represent the same point in Hilb_*Q*_(2*y* + 2). In general, *𝒪*_*L*_^⊕*k*^ and *𝒪*_*kL*_ with *k* ≥ 1 represent the same point in **M**(0, *k*, *k*). Thus, *𝒪*_*L*_ with *L* ∈ |*𝒪*_*Q*_(*n*, 0)| is strictly semistable if and only if *n* ≥ 2. Conversely, let us choose a semistable sheaf *ℱ* with *χ*_*ℱ*_(*x*, *y*) = *ny* + *n*. In particular, the schematic support *L* = Supp(*ℱ*) of *ℱ* is in |*𝒪*_*Q*_(*n*, 0)|. Since *χ*(*ℱ*) = *n* > 0, there exists a nontrivial morphism *𝒪*_*Q*_ → *ℱ* and it induces an injection *𝒪*_*L*_1__ → *ℱ*, where *L*_1_ is a subscheme of *L*. Here we have *L*_1_ ∈ |*𝒪*_*Q*_(*s*, 0)| for some *s* ≤ *n* and so *χ*_*L*_1__(*x*, *y*) = *sx* + *s*. Thus, the quotient *𝒢* = *ℱ*/*𝒪*_*L*_1__ is a semistable sheaf with *χ*_*𝒢*_(*x*, *y*) = (*n* − *s*)*y* + (*n* − *s*). By induction, we have [*𝒢*] = [*𝒪*_*L*_2__] with *L*_2_ ∈ |*𝒪*_*Q*_(*n* − *s*, 0)|. In particular, *ℱ* is an extension of *𝒪*_*L*_2__ by *𝒪*_*L*_1__ with *L*_1_ + *L*_2_ ∈ |*𝒪*_*Q*_(*n*, 0)| and thus *ℱ* is equivalent to *𝒪*_*L*_1__ ⊕ *𝒪*_*L*_2__.

Now, let us assume that 0 < *t* < *n* and fix *ℱ* ∈ **M**(0, *n*, *t*) with *C* : = *C*_*ℱ*_ ∈ |*𝒪*_*C*_(*n*, 0)|. Since *χ*(*ℱ*) = *t* > 0, there is a nonzero map *f* : *𝒪*_*Q*_ → *ℱ*. Since *ℱ* is an *O*_*C*_-sheaf, *f* induces a nonzero map *h* : *𝒪*_*C*_ → *ℱ*. Since *𝒪*_*C*_ has slope 1 > *t*/*n* and it is semistable, we get a contradiction. Alternatively, as in Lemma 4.10 of [6], we may first take the schematic support *T*⊆*C* of *Im*⁡(*h*) and then use an injective map *𝒪*_*T*_ → *ℱ* with *𝒪*_*T*_ ∈ |*𝒪*_*Q*_(*n*′, 0)| with 1 ≤ *n*′ < *n*, and thus we have *μ*(*𝒪*_*T*_) = 1.

For the case of *m* = 1, that is, *χ*_*ℱ*_(*x*, *y*) = *x* + *ny* + *t*, it is enough to check the case *t* = 1 since gcd(1, *n*) = 1.

**Proposition 11**

**M**(1, *n*, 1) consists of *𝒪*_*C*_ with *C* ∈ |*𝒪*_*Q*_(*n*, 1)|. In particular, one has **M**(1, *n*, 1)≅*ℙ* ^2*n*+1^.

**Proof**

From the sequence

(28) $$\begin{array}{c} 0⟶{𝒪}_{Q}\left(-n,-1\right)⟶{𝒪}_{Q}⟶{𝒪}_{C}⟶0, \end{array}$$

we have *χ*_*𝒪*_*C*__(*x*, *y*) = *x* + *ny* + 1 and *𝒪*_*C*_ is semistable by Lemma 9. Conversely, let *ℱ* be a semistable sheaf with *χ*_*ℱ*_(*x*, *y*) = *x* + *ny* + 1 and so *C* : = *C*_*ℱ*_ is a curve in |*𝒪*_*Q*_(*n*, 1)|. Since we have *χ*(*ℱ*) = 1, there exists a nonzero map *𝒪*_*Q*_ → *ℱ* and it induces a nonzero map *h* : *𝒪*_*C*_ → *ℱ*. Note that *Im*⁡(*h*) has no 0-dimensional torsion since *ℱ* is semistable. Since *𝒪*_*C*_ is also semistable, we have

(29) p(𝒪C)≤p(Im⁡(h))≤p(ℱ).

The map *h* factors through an injection *𝒪*_*D*_↪*ℱ*, where *D* is a curve contained in *C*. If *D* is properly contained in *C*, we have *p*(*𝒪*_*D*_) > *p*(*ℱ*) contradicting the semistability of *ℱ* and thus we have *D* = *C*; that is, *h* is an isomorphism from *𝒪*_*C*_ to its image. Since *𝒪*_*C*_ and *ℱ* have the same Hilbert polynomial, we have *ℱ*≅*𝒪*_*C*_.

## 3. Hilbert Bipolynomial 2*x* + 2*y* + 1

For the moduli space of semistable sheaves with linear Hilbert bipolynomial 2*x* + 2*y* + *t*, it is enough to investigate the case when *t* = 1,2. Let us denote the moduli space **M**(2,2, *t*) by **M**_*t*_.

**Proposition 12**

The moduli space **M**_1_ consists of the unique nontrivial extensions *ℱ* of *𝒪*_*P*_ by *𝒪*_*C*_ for each curve *C* ∈ |*𝒪*_*Q*_(2,2)| and a point *P* ∈ *C*, and one also has *h* ^0^(*ℱ*) = 1.

**Proof**

Since *χ*(*ℱ*) = 1, there is a nonzero map *𝒪*_*Q*_ → *ℱ*, inducing a nonzero map *h* : *𝒪*_*C*_ → *ℱ*, where *C* : = *C*_*ℱ*_ ∈ |*𝒪*_*Q*_(2,2)|. Since *χ*_*𝒪*_*C*__(*x*, *y*) = 2*x* + 2*y*, we have *p*(*𝒪*_*C*_) = 0 < 1/4 = *p*(*ℱ*). The map *h* factors through an injection *𝒪*_*D*_↪*ℱ*, where *D* is a curve contained in *C*. If *D* is properly contained in *C*, we have *p*(*𝒪*_*D*_) > *p*(*ℱ*) contradicting to the semistability of *ℱ* and thus we have *D* = *C*; that is, *h* is an isomorphism from *𝒪*_*C*_ to its image, that is, we have

(30) $$\begin{array}{c} 0⟶{𝒪}_{C}⟶ℱ⟶𝒢⟶0, \end{array}$$

where *χ*_*𝒢*_(*x*, *y*) = 1. In particular, we have *𝒢*≅*𝒪*_*P*_, the skyscraper sheaf supported on a point *P* ∈ *C*. Since *ℱ* has no 0-dimensional torsion, the sequence does not split. Note that Ext^1^(*𝒪*_*P*_, *𝒪*_*Q*_)≅*H* ^1^(*𝒪*_*P*_)^∨^ = 0, and thus from the sequence of *C* we have

(31) 0⟶Ext1(𝒪P,𝒪C)⟶Ext2(𝒪P,𝒪Q(−2,−2))⟶sExt2(𝒪P,𝒪Q).

Here, the map *s* is the transpose of Hom(*𝒪*_*Q*_(2,2), *𝒪*_*P*_) → Hom(*𝒪*_*Q*_, *𝒪*_*P*_) which is given by the multiplication by the defining equation of *C*. Since *P* is a point on *C*, the map *s* is a zero map. In particular, the dimension of Ext^1^(*𝒪*_*P*_, *𝒪*_*C*_) is 1 and so *ℱ* corresponds to a unique nontrivial extension

(32) $$\begin{array}{c} 0⟶{𝒪}_{C}⟶ℱ⟶{𝒪}_{P}⟶0. \end{array}$$

From the sequence (32), we have *h* ^0^(*ℱ*) ≤ 2 and that *h* ^0^(*ℱ*) = 1 if and only if no injective map *𝒪*_*C*_ → *ℱ* is an isomorphism at *P*. This is certainly true if *ℱ* is not locally free of rank 1 at *P*. Note that *ℱ* is a line bundle at each point of *C*∖{*P*} and thus it is sufficient to prove *h* ^0^(*ℱ*) = 1 when *ℱ* is a line bundle on the curve *C*. In this case the nonexistence of a section of *ℱ* that does not vanish at *P* is equivalent to the nonsplitting of (32). Thus, we have *h* ^0^(*ℱ*) = 0 and so the point *P* is uniquely determined by *ℱ*.

Conversely, let us assume that *ℱ* is a nontrivial extension of *𝒪*_*P*_ by *𝒪*_*C*_, where *P* is a point on *C*. If *ℱ* is not semistable, then there exists a subsheaf *𝒦* ⊂ *ℱ* with *p*(*𝒦*) > *p*(*ℱ*) = 1/4 and so we have *χ*_*𝒦*_(*x*, *y*) = *m*′*x* + *n*′*y* + *t*′ with (*m*′, *n*′)≤(2,2) and *t*′ ≥ 1. If the composite *s* : *𝒦* → *ℱ* → *𝒪*_*P*_ is a zero map, then we have an injection *𝒦*↪*𝒪*_*C*_, contradicting the semistability of *𝒪*_*C*_. Thus, the composite is surjective and so we have the following diagram:

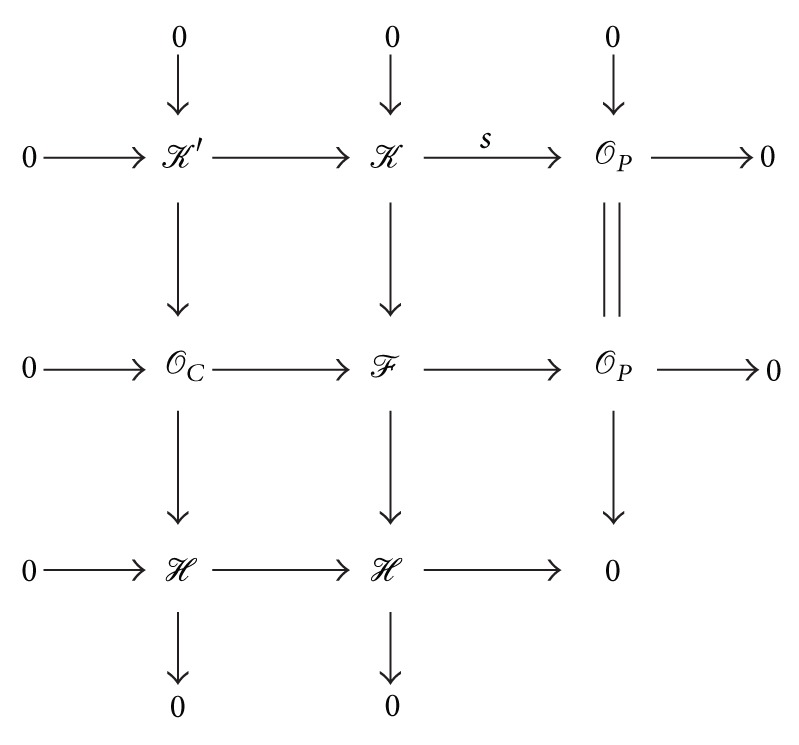
(33)
Here, *𝒦*′ is the kernel of the map *s* and *ℋ* is the quotient *ℱ*/*𝒦*. Since *χ*_*𝒦*′_(*x*, *y*) = *m*′*x* + *n*′*y* + (*t*′ − 1) and *𝒪*_*C*_ is semistable, we have *t*′ = 1 and thus *χ*_*ℋ*_(*x*, *y*) = (2 − *m*′)*x* + (2 − *n*′)*y* with no constant term. Since *ℋ* is the quotient of *𝒪*_*C*_, it must be *𝒪*_*T*_ for some curve *T* contained in *C*. But no such curves have the Hilbert polynomials with no constant term. Hence *ℱ* is semistable.

**Remark 13**

There is no strictly semistable sheaf in **M**_1_. Let us assume the existence of a polystable sheaf *ℱ* = *ℱ*_1_ ⊕ ⋯⊕*ℱ*_*s*_ with *s* ≥ 2. We have *χ*(*ℱ*) = 1 = *χ*(*ℱ*_1_)+⋯+*χ*(*ℱ*_*s*_). If we let *χ*_*ℱ*_*i*__(*x*, *y*) = *a*_*i*_ *x* + *b*_*i*_ *y* + *c*_*i*_, then we have

(34) $$\begin{array}{c} c_{1}+\cdots +c_{s}=1,\;\;\frac{c_{i}}{a_{i}+b_{i}}=\frac{1}{2},\;\forall i. \end{array}$$

It implies that *c*_*i*_ > 0 for all *i* and thus we have *s* = 1, a contradiction.

**Proposition 14**

A sheaf *ℱ* is in **M**_1_ if and only if it admits the following resolution:

(35) $$\begin{array}{c} 0⟶𝒜\overset{\varphi }{⟶}{𝒪}_{Q}⊕{𝒪}_{Q}\left(-1,-1\right)\overset{\psi }{⟶}ℱ⟶0, \end{array}$$

where *𝒜* : = *𝒪*_*Q*_(−2, −1) ⊕ *𝒪*_*Q*_(−1, −2) and $\varphi =\left(\begin{array}{cc} h_{1} & l_{1}\\ h_{2} & l_{2} \end{array}\right)$. Here, *f* : = *h*_1_ *l*_2_ − *h*_2_ *l*_1_ is a defining equation of *C*_*ℱ*_.

**Proof**

Note that *h* ^0^(*ℱ*) = 1 and so *h* ^1^(*ℱ*) = 0. If *ℱ* admits the sequence (32), then *ℱ* is globally generated outside *P* and so is *ℱ*(1,1). Take any *A* ∈ |*𝒪*_*Q*_(1,1)| which is not contained in *C* and with *P* ∉ *A*. The multiplication by an equation of *A* gives an exact sequence

(36) $$\begin{array}{c} 0⟶ℱ⟶ℱ\left(1,1\right)⟶{ℱ(1,1)|}_{A}⟶0, \end{array}$$

where deg⁡(*ℱ*(1,1)|_*A*_) = deg⁡(*A*∩*C*) = 4. Thus we have *h* ^1^(*ℱ*(1,1)) = 0 and *h* ^0^(*ℱ*(1,1)) = 5. Together with the exact sequence (32) tensored by *𝒪*_*Q*_(1,1), we obtain that *ℱ*(1,1) is globally generated at *P* and so we have a surjection

(37) $$\begin{array}{c} \psi :{𝒪}_{Q}⊕{𝒪}_{Q}\left(-1,-1\right)⟶ℱ⟶0. \end{array}$$

Let us set *ℋ* : = *ker*⁡(*ψ*) and then *ℋ* is a torsion-free sheaf of rank 2 on *Q* with *c*_1_ = (−3, −3). By Theorem 19.9 in [7], the sheaf *ℋ* is locally free. Note that *χ*_*ℋ*(1,2)_(*x*, *y*) = 2*xy* + 3*x* + *y* + 1. Thus, we have *h* ^0^(*ℋ*(1,2)) > 0 and so we have an exact sequence

(38) $$\begin{array}{c} 0⟶{𝒪}_{Q}\left(a,b\right)⟶ℋ\left(1,2\right)⟶{ℐ}_{Z}\left(-1-a,1-b\right)⟶0, \end{array}$$

where *Z* is a 0-dimensional subscheme of *Q* and (*a*, *b*)∈{(0,0), (0,1)}. If (*a*, *b*) = (0,1), then we have *χ*_*ℐ*_*Z*_(−1,0)_(*x*, *y*) = *xy* + *x* + 1 and it is absurd since *χ*_*𝒪*_*Q*_(−1,0)_(*x*, *y*) = *xy* + *x*. Thus, we have (*a*, *b*) = (0,0) and *Z* = *∅*. Since Ext^1^(*𝒪*_*Q*_(−1,1), *𝒪*_*Q*_) = 0, we have *ℋ*(1,2)≅*𝒪*_*Q*_ ⊕ *𝒪*_*Q*_(−1,1) and the sequence (35). Note that the map *φ* : *ℋ* → *𝒪*_*Q*_ ⊕ *𝒪*_*Q*_(−1, −1) is given by $\left(\begin{array}{cc} h_{1} & l_{1}\\ h_{2} & l_{2} \end{array}\right)$, where *f* : = *h*_1_ *l*_2_ − *h*_2_ *l*_1_ is a defining equation of *C* = *C*_*ℱ*_.

The converse is trivial.

**Remark 15**

Using the proof of Lemma 5.3 in [2], we can obtain the same assertion of Proposition 14. Similarly, we also obtain that *ℱ*(1,0) is globally generated and so a surjection *φ*′ : *𝒪*_*Q*_ ⊕ *𝒪*_*Q*_(−1,0) → *ℱ*. In this case, *ker*⁡(*φ*′) is no longer a direct sum of two line bundles.

Let us define a vector space *W* to be

(39) $$\begin{array}{c} W:=\text{Hom}\left(𝒜,{𝒪}_{Q}⊕{𝒪}_{Q}\left(-1,-1\right)\right) \end{array}$$

and *W* ^0^ ⊂ *W* to be the set of *φ* ∈ *W* such that *h*_1_ *l*_2_ − *h*_2_ *l*_1_ ≠ 0. Then we have a surjective morphism

(40) $$\begin{array}{c} \pi :W^{0}⟶M_{1}. \end{array}$$

Let us choose *φ*_1_, *φ*_2_ ∈ *W* ^0^ with *π*(*φ*_1_) = *π*(*φ*_2_); that is, we have the following diagram:

(41) 0⟶𝒜⟶φ1𝒪Q⊕𝒪Q(−1,−1)⟶ψ1ℱ⟶0              ↓f0⟶𝒜⟶φ2𝒪Q⊕𝒪Q(−1,−1)⟶ψ2ℱ⟶0,

where *f* is an isomorphism. Since Ext^1^(*𝒪*_*Q*_ ⊕ *𝒪*_*Q*_(−1, −1), *𝒜*) = 0, we have a map *f*_1_ ∈ End(*𝒪*_*Q*_ ⊕ *𝒪*_*Q*_(−1, −1)) associated with *f*. Note that *f*_1_ is given by $\left(\begin{array}{cc} a & 0\\ z & b \end{array}\right)$, where *a*, *b* ∈ *ℂ* ^×^ and *z* ∈ *H* ^0^(*𝒪*_*Q*_(1,1)). Similarly, we have a map *f*_2_ : *𝒜* → *𝒜* which is $\left(\begin{array}{cc} c_{1} & 0\\ 0 & c_{2} \end{array}\right)$, where *c*_1_, *c*_2_ ∈ *ℂ* ^×^. In particular, we have

(42) $$\begin{array}{c} \varphi_{2}={\left(\begin{array}{cc} c_{1} & 0\\ 0 & c_{2} \end{array}\right)}^{-1}\varphi_{1}\left(\begin{array}{cc} a & 0\\ z & b \end{array}\right). \end{array}$$

In this equation, we can assume that *c*_1_ = 1. In other words, *π*(*φ*_1_) = *π*(*φ*_2_) if and only if *φ*_1_ and *φ*_2_ are in the same orbit in *W* ^0^ under the action by

(43) G:=(Aut(𝒜)×Aut(𝒪Q⊕𝒪Q(−1,−1)))ℂ×={((100c),(a0zb)) ∣ a,b,c∈ℂ×, z∈H0(𝒪Q(1,1))}.

**Theorem 16**

*π* : *W* ^0^ → **M**_1_ is a geometric quotient map by the action of **G**. In particular, one has **M**_1_≅*W* ^0^/**G** and so **M**_1_ is isomorphic to *𝒫ic*_(2,2)_^1^.

**Proof**

To get the assertion it suffices to prove that it has local sections as in Lemma 5.1 and Theorem 5.5 in [3].

Since every element of **M**_1_ is stable, **M**_1_ has a universal family *ℍ*_1_ on **M**_1_ × *ℙ* ^2^ (see page 180 of [8] or Theorem 4.6.5 of [9]). Since every semistable sheaf with bipolinomial 2*x* + 2*y* + 5 is of the form *ℱ*(1,1) for a unique *ℱ* ∈ **M**_1_, we also have a universal family *ℍ*_5_ on **M**_5_ × *ℙ* ^2^ with *ℱ*(1,1) as the fibre. Since *h* ^0^(*ℱ*) = 1 and *h* ^1^(*ℱ*) = 0 for all *ℱ* ∈ **M**_1_, the base change theorem gives that *u*_∗_(*ℍ*_1_) is a line bundle on **M**_1_, where *u* : **M**_1_ × *ℙ* ^2^ → *M*_1_ is the first projection. Since *h* ^0^(*ℱ*(1,1)) = 5 and *h* ^1^(*ℱ*(1,1)) = 0 for all *ℱ* ∈ **M**_1_, the base change theorem gives that *v*_∗_(*ℍ*_5_) is a vector bundle of rank 5 on **M**_1_ by identifying **M**_5_ with **M**_1_, where *v* : **M**_5_ × *ℙ* ^2^ → *M*_5_. For a fixed *ℱ* ∈ **M**_1_ and a matrix *φ* ∈ *π* ^−1^(*ℱ*), let us write $\varphi =\left(\begin{array}{cc} h_{1} & l_{1}\\ h_{2} & l_{2} \end{array}\right)$, where *f* : = *h*_1_ *l*_2_ − *h*_2_ *l*_1_ is a defining equation of *C*_*ℱ*_. Take an open neighborhood *U* of *ℱ* in **M**_1_ over which *u*_∗_(*ℍ*_1_) and *v*_∗_(*ℍ*_5_) are trivial. The matrix *φ* was constructed starting with a section *σ* of *ℱ*(1,1) which spans *ℱ*(1,1) together with the twist *σ*′ of a nonzero section of *ℱ*. Since *u*_∗_(*ℍ*_1_)|_*U*_ and *v*_∗_(*ℍ*_5_)|_*U*_ are trivial, there are maps *e*_1_ : *𝒪*_*U*_ → *𝒪*_*U*_ and *e*_2_ : *𝒪*_*U*_ → *𝒪*_*U*_^⊕5^ with *e*_1_(*ℱ*) = *σ*′ and *e*_2_(*ℱ*) = *σ*. Since *σ*′ and *σ* span *ℱ*, there is a neighborhood *V* of *ℱ* in *U* such that the sections *e*_1_(*𝒢*) and *e*_2_(*𝒢*) span every *𝒢* ∈ *V*. The construction of *φ* gives that *e*_1_ and *e*_2_ induce a section of *π* in a neighborhood of *φ* whose image by *π* is *V*.

As an automatic consequence, we obtain that **M**_1_ is irreducible and unirational. In fact, we can prove more.

**Theorem 17**

**M**_1_ is rational.

**Proof**

Let Δ ⊂ *Q* × *Q* be the diagonal and denote its ideal sheaf by *ℐ*_Δ_. Denoting by *p*_1_ and *p*_2_ the projection from *Q* × *Q* to each factor, let us define a sheaf *𝒰* to be *p*_1_*𝒪_*Q*_(2,2))⊠*ℐ*_Δ_ on *Q* × *Q*. For each point *P* ∈ *Q*, we have *𝒰*|_*Q*×{*P*}_≅*ℐ*_*P*_(2,2). Thus, we have *h* ^1^(*𝒰*|_*Q*×{*P*}_) = 0 and so *p*_2__∗_ *𝒰* is a vector bundle of rank 8 on *Q* since *h* ^0^(*𝒰*|_*Q*×{*P*}_) = 8. Let us consider the projective bundle

(44) $$\begin{array}{c} 𝒵=\mathbb{P}\left({p_{2}}_{*}𝒰\right)⟶Q. \end{array}$$

By its definition, the fibre of *𝒵* over a point *P* ∈ *Q* is the set of curves of type (2,2) on *Q* passing through *P* and so there is a natural map from *𝒵* to *ℙH* ^0^(*𝒪*_*Q*_(2,2))≅*ℙ* ^8^. In other words, *𝒵* is the universal curve of type (2,2) on *Q* and it is isomorphic to **M**_1_. Since *𝒵* is locally trivial over *Q*, it is rational.

## 4. Hilbert Bipolynomial 2*x* + 2*y* + 2

**Lemma**

Any sheaf *ℱ* ∈ **M**_2_ admits one of the following types*:*

- 0 → *𝒪*_*C*_ → *ℱ* → *η* → 0, where *η* is a skyscraper *𝒪*_*C*_-sheaf with degree 2,
- 0 → *𝒪*_*T*_1__ → *ℱ* → *𝒪*_*T*_2__ → 0 with *T*_1_, *T*_2_ ∈ |*𝒪*_*Q*_(1,1)|,
- 0 → *𝒪*_*T*_1__ → *ℱ* → *𝒪*_*T*_2__ → 0, where *T*_1_ ∈ |*𝒪*_*Q*_(*a*, *b*)| and *T*_2_ ∈ |*𝒪*_*Q*_(2 − *a*, 2 − *b*)| with (*a*, *b*) ∈ {(1,2), (2,1)}.

**Proof**

Since *χ*(*ℱ*) = 2, we have *h* ^0^(*ℱ*) ≥ 2. Thus, there exists a nonzero map *𝒪*_*Q*_ → *ℱ* and it induces a nonzero map *h* : *𝒪*_*C*_ → *ℱ*, where *C* : = *C*_*ℱ*_ ∈ |*𝒪*_*Q*_(2,2)|. The map *h* factors through an injection *𝒪*_*T*_1__↪*ℱ* where *T*_1_ is a curve contained in *C*:

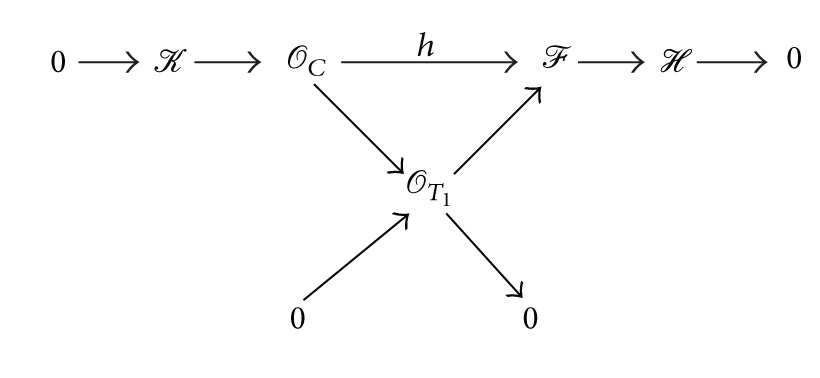
(45)
If we have *T*_1_ = *C*, that is the map *h* is an isomorphism from *𝒪*_*C*_ to its image in *ℱ*, then its cokernel *ℋ* is the skyscraper sheaf supported on two points, say *P*_1_, *P*_2_ ∈ *C*. Thus we have the sequence

(46) $$\begin{array}{c} 0⟶{𝒪}_{C}⟶ℱ⟶\eta ⟶0. \end{array}$$

Let us assume that *T*_1_ is properly contained in *C* and then we obtain that *T*_1_ has bidegree (1,1), (1,2), or (2,1) since *p*(*𝒪*_*T*_1__) ≤ *p*(*ℱ*) = 1/2 and *ℱ* is semistable. Let *T*_2_ ⊂ *Q* be the only curve such that *T*_1_ + *T*_2_ = *C*. Let *ℋ*′ be the quotient of *ℋ* by its torsion *τ*, that is, *ℋ*′ : = *ℋ* ^*DD*^.

First, assume *T*_1_ ∈ |*𝒪*_*Q*_(1,1)| and so we have *χ*_*ℋ*′_(*x*, *y*) = *x* + *y* + 1 − deg⁡(*τ*). Since *ℱ* is semistable, we get *τ* = 0. Since every quotient of *ℱ* has the slope at least 1/2, the same is true for *ℋ*. Thus, *ℋ* is semistable and Proposition 11 gives *ℋ*≅*𝒪*_*T*_2__.

Now, without loss of generality, let us assume that *T*_1_ ∈ |*𝒪*_*Q*_(1,2)|, that is, *χ*_*𝒪*_*T*_1___(*x*, *y*) = 2*x* + *y* + 1 and so we have *χ*_*ℋ*_(*x*, *y*) = *y* + 1. If *ℋ* has 0-dimensional torsion *𝒯* with length *k* ≥ 1, then the quotient *ℋ*/*𝒯* is a quotient of *ℱ* with the *p*-slope 1 − *k* ≤ 0, contradicting the semistability of *ℱ*. Thus *ℋ* has no 0-dimensional torsion and so we have *ℋ*≅*𝒪*_*T*_2__ for a curve *T*_2_ ∈ |*𝒪*_*Q*_(1,0)| with *C* = *T*_1_ + *T*_2_.

**Corollary 19**

Every sheaf in **M**_2_ is globally generated.

**Proof**

Let us take *ℱ* ∈ **M**_2_ and then there is no nonzero map *ℱ* → *𝒪*_*C*_≅*ω*_*C*_ since *ℱ* is semistable. Thus we have *h* ^1^(*ℱ*) = 0 and so *h* ^0^(*ℱ*) = 2. It is clear that *ℱ* of types (B) and (C) is globally generated and so we may assume that *ℱ* is of type (A), but neither of (B) nor of (C).

Let *ℋ*⊆*ℱ* be the image of the evaluation map *H* ^0^(*ℱ*) ⊗ *𝒪*_*Q*_ → *ℱ* and then *ℋ* is pure. Assume that *ℋ* ≠ *ℱ*. Since *ℱ* is of type (A), it is globally generated outside at most two points of *C*_red_. In particular, we have *χ*_*ℋ*_(*x*, *y*) = 2*x* + 2*y* + *c* with *c* ≤ 1 and deg⁡(*ℱ*/*ℋ*) = 2. Since *h* ^0^(*ℋ*) = *h* ^0^(*ℱ*) = 2, we have *h* ^1^(*ℋ*) = 2 − *c*. Note that every nonzero section of *ℋ* vanishes at finitely many points since *ℱ* is neither of types (B) nor (C). Since *h* ^0^(*𝒪*_*C*_) < *h* ^0^(*ℋ*), we have *ℋ* ≠ *𝒪*_*C*_ and *c* = 1. A nonzero section of *ℋ* induces an exact sequence

(47) $$\begin{array}{c} 0⟶{𝒪}_{C}⟶ℋ⟶𝒢⟶0, \end{array}$$

where *𝒢*≅*𝒪*_*P*_ for some *P* ∈ *C*_red_. Since *ℋ* is pure, this exact sequence does not split. As in the proof of Proposition 12, we get a contradiction. Thus, we have *ℋ* = *ℱ* and so *ℱ* is globally generated.

**Lemma 20**

*ℱ* is a sheaf in **M**_2_ if and only if it admits a sequence

(48) $$\begin{array}{c} 0⟶{𝒪}_{Q}{\left(-1,-1\right)}^{⊕2}\overset{\varphi }{⟶}{𝒪}_{Q}^{⊕2}⟶ℱ⟶0, \end{array}$$

where $\varphi =\left(\begin{array}{cc} z_{11} & z_{12}\\ z_{21} & z_{22} \end{array}\right)$, *z*_*ij*_ ∈ *H* ^0^(*𝒪*_*Q*_(1,1)) such that *f* : = *z*_11_ *z*_22_ − *z*_12_ *z*_21_ is a defining equation of *C*_*ℱ*_.

**Proof**

Let *ℱ* ∈ **M**_2_ be a sheaf of type (A) and then it is globally generated by Corollary 19. Since *h* ^0^(*ℱ*) = 2, we have a surjection

(49) $$\begin{array}{c} \psi :{𝒪}_{Q}^{⊕2}⟶ℱ⟶0. \end{array}$$

Let us set *ℋ* : = *ker*⁡(*ψ*) and then it is a torsion-free sheaf of rank 2 on *Q* with *c*_1_ = (−2, −2). By Theorem 19.9 in [7], *ℋ* is locally free. Note that *h* ^0^(*ℋ*(1,1)) = 2. From the sequence

(50) $$\begin{array}{c} 0⟶ℋ\left(1,0\right)⟶{𝒪}_{Q}{\left(1,0\right)}^{⊕2}⟶ℱ\left(1,0\right)⟶0, \end{array}$$

we obtain that the map *H* ^0^(*𝒪*_*Q*_(1,0)^⊕2^) → *H* ^0^(*ℱ*(1,0)) is an isomorphism and so *h* ^1^(*ℋ*(1,0)) = 0. Similarly, we have *h* ^1^(*ℋ*(0,1)) = 0 and *h* ^2^(*ℋ*) = *h* ^1^(*ℱ*) = 0. By Remark 2.3 in [10], we obtain that *ℋ*(1,1) is globally generated. Since *c*_1_(*ℋ*(1,1)) = 0 or *h* ^0^(*ℋ*(1,1)) = 2, we have *ℋ*≅*𝒪*_*Q*_(−1, −1)^⊕2^ and the resolution (48). The cases of the other types also work verbatim.

**Definition 21**

Let us define a subscheme *𝔄* ⊂ **M**_2_ as follows*: *

(51) 𝔄:={ℱ∈M2 ∣ ℱ  admits  a  nontrivial   extension  of  type  (A)}.

Similarly, we define *𝔅* and *ℭ* for the semistable sheaves of types (B) and (C), respectively. In particular, we have

(52) $$\begin{array}{c} M_{2}=𝔄\cup 𝔅\cup ℭ. \end{array}$$

**Lemma 22**

The sheaves *ℱ* of type (*B*) are strictly semistable. In particular, they are contained in *𝔅*.

**Proof**

It is enough to check the semistability of *ℱ*. Let *𝒦* be a subsheaf of *ℱ* with *p*(*𝒦*) > 1/2 and the quotient sheaf *ℋ* : = *ℱ*/*𝒦*. If the composite map *s* : *𝒦*↪*ℱ*↠*𝒪*_*T*_2__ is a zero map, then *𝒦* is a subsheaf of *𝒪*_*T*_1__, contradicting the semistability of *𝒪*_*T*_1__. The sheaf *Im*⁡(*s*) is a subsheaf of *𝒪*_*T*_2__ and so we have *p*(*Im*⁡(*s*)) ≤ 1/2. Similarly, the sheaf *ker*⁡(*s*) is a subsheaf of *𝒪*_*T*_1__ and so *p*(*ker*⁡(*s*)) ≤ 1/2. From the exact sequence

(53) 0⟶ker⁡(s)⟶𝒦⟶sIm⁡(s)⟶0,

we have *p*(*𝒦*) ≤ 1/2, a contradiction.

Let us denote by ∂**M**_2_ the closed subscheme of **M**_2_, consisting of the strictly semistable sheaves.

**Corollary 23**

One has

(54) $$\begin{array}{c} \partial M_{2}=𝔅≅\frac{\left({\mathbb{P}}^{3}\times {\mathbb{P}}^{3}\right)}{{𝔖}_{2}}, \end{array}$$

where *𝔖*_2_ is the permutation group of order 2. In particular, ∂**M**_2_ is a rational variety.

**Proof**

Obviously, we have *𝔅* ⊂ ∂**M**_2_. Let *ℱ* be a strictly semistable sheaf and so it has a proper quotient sheaf *ℋ* with *p*(*ℋ*) = 1/2. From the semistability of *ℱ*, *ℋ* has no 0-dimensional torsion. From the equality *p*(*ℱ*) = *p*(*ℋ*), we obtain that *ℋ* is also semistable. Since *p*(*ℋ*) = 1/2, the Hilbert bipolynomial of *ℋ* is either 2*x* + 1, 2*y* + 1 or *x* + *y* + 1. The first 2 cases cannot happen due to Proposition 10. Thus, we have *χ*_*ℋ*_(*x*, *y*) = *x* + *y* + 1 and so *ℋ*≅*𝒪*_*T*_2__ with *T*_2_ ∈ |*𝒪*_*Q*_(1,1)| and *T*_2_ ⊂ *C*_*ℱ*_ by Proposition 11. If *𝒦* is the kernel of the quotient map *ℋ* → *ℋ*, then its p-slope is again 1/2 and so *𝒦* is semistable. Similarly as before, we have *𝒦*≅*𝒪*_*T*_1__ with *T*_1_ ∈ |*𝒪*_*Q*_(1,1)| and *C*_*ℱ*_ = *T*_1_ + *T*_2_. Hence, we have *ℱ* ∈ *𝔅*.

Let *ℱ* be a sheaf of type (B), that is, it corresponds to a pair of two curves {*T*_1_, *T*_2_}. Let us assume that *ℱ* admits another sequence

(55) $$\begin{array}{c} 0⟶{𝒪}_{T_{3}}⟶ℱ⟶{𝒪}_{T_{4}}⟶0 \end{array}$$

with *T*_3_, *T*_4_ ∈ |*𝒪*_*Q*_(1,1)|. Note that *𝒪*_*T*_*i*__ is stable for all *i*. Thus, the composite map *s* : *𝒪*_*T*_3__ → *ℱ* → *𝒪*_*T*_2__ is either a zero map or an isomorphism. In the former case, we have *𝒪*_*T*_3__≅*𝒪*_*T*_1__ and so *𝒪*_*T*_4__≅*𝒪*_*T*_2__. In the latter case, we have *𝒪*_*T*_3__≅*𝒪*_*T*_2__ and *𝒪*_*T*_4__≅*𝒪*_*T*_1__. Hence, the class of a strictly semistable sheaf *ℱ* corresponds to a uniquely determined pair of two curves in |*𝒪*_*Q*_(1,1)| and we have *𝔅*≅(*ℙ* ^3^ × *ℙ* ^3^)/*𝔖*_2_. The second assertion follows from the fact that any symmetric product *S* ^*d*^(*ℙ* ^*N*^) of any projective space is a rational variety (see Theorems 4.2.8 and 4.2.8′ in page 137 of [11, 12]).

**Lemma 24**

For two curves *T*_1_, *T*_2_ ∈ |*𝒪*_*Q*_(1,1)|, one has

(56) $$\begin{array}{c} \dim{\mathrm{Ext}}^{1}\left({𝒪}_{T_{2}},{𝒪}_{T_{1}}\right)=\{\begin{array}{cc} 3, & if\;\;T_{1}=T_{2};\;\\ 2, & if\;\;T_{1}\neq T_{2}.\; \end{array} \end{array}$$

**Proof**

Note that we have

(57) $$\begin{array}{c} \text{Ex}{\text{t}}^{2}\left({𝒪}_{T_{2}},{𝒪}_{Q}\left(-1,-1\right)\right)=H^{0}{\left({𝒪}_{T_{2}}\left(-1,-1\right)\right)}^{\vee }=0. \end{array}$$

Thus, if we apply the functor Hom(*𝒪*_*T*_2__, −) to the sequence of *T*_1_, we obtain

(58) 0⟶Hom(𝒪T2,𝒪T1)⟶Ext1(𝒪T2,𝒪Q(−1,−1))⟶Ext1(𝒪T2,𝒪Q)⟶Ext1(𝒪T2,𝒪T1)⟶0.

We also have

(59) $$\begin{array}{c} \text{Ex}{\text{t}}^{1}\left({𝒪}_{T_{2}},{𝒪}_{Q}\left(-1,-1\right)\right)≅H^{1}{\left({𝒪}_{T_{2}}\left(-1,-1\right)\right)}^{\vee }≅H^{0}\left({𝒪}_{T_{2}}\right)\\ \text{Ex}{\text{t}}^{1}\left({𝒪}_{T_{2}},{𝒪}_{Q}\right)≅H^{1}{\left({𝒪}_{T_{2}}\left(-2,-2\right)\right)}^{\vee }≅H^{0}\left({𝒪}_{T_{2}}\left(1,1\right)\right) \end{array}$$

and so their dimensions are 1 and 3, respectively. As *𝒪*_*Q*_-sheaves, we have

(60) $$\begin{array}{c} h^{0}\left(ℋ\text{om}\left({𝒪}_{T_{2}},{𝒪}_{T_{1}}\right)\right)=\{\begin{array}{cc} 1, & \text{if}\;\;T_{1}=T_{2};\;\\ 0, & \text{otherwise}, \end{array} \end{array}$$

for example, because *T*_1_ and *T*_2_ are reduced, and so the assertion is derived.

**Lemma 25**

The sheaves *ℱ* of type (*C*), but not of type (*B*), are stable. In particular, the sheaves of type (*C*) are semistable.

**Proof**

As before let us assume the existence of a proper subsheaf *𝒦* of *ℱ* with *p*(*𝒦*) ≥ 1/2 and the quotient sheaf *ℋ* : = *ℱ*/*𝒦*. Since the composite *s* : *𝒦*↪*ℱ*²↠*𝒪*_*T*_2__ is not a zero map, thus we have *Im*⁡(*s*)≅*𝒪*_*T*_2__(−*Z*) for a 0-dimensional subscheme *Z* of *T*_2_ with length *k*. In particular, its Hilbert bipolynomial is *y* + 1 − *k*. If we let *χ*_*𝒦*_(*x*, *y*) = *m*′*x* + *n*′*y* + *t*′, then we have *p*(*𝒦*) = *t*′/(*m*′ + *n*′) ≥ 1/2. In particular, we have *t*′ ≥ 1. If we define *𝒦*′ to be the kernel of the map *s*, then it is a subsheaf of *𝒪*_*T*_1__ and thus we have *p*(*𝒦*′) = (*t*′ − 1 + *k*)/(*m*′ + *n*′ − 1) ≤ 1/3. Combining the two inequalities, we have *k* = 0 and so the map *s* is surjective. Thus, we have *ℋ*≅*𝒪*_*T*_1__/*𝒦*′. Note also that *t*′ can be either 1 or 2. If *t*′ = 2, then we have *m*′ = *n*′ = 2 and so *χ*_*𝒦*′_(*x*, *y*) = 2*x* + *y* + 1 = *χ*_*𝒪*_*T*_1___(*x*, *y*). In particular, we have *ℋ* = 0 and so *𝒦*≅*ℱ*, a contradiction. Now, assume *t*′ = 1 and so *m*′ + *n*′ ≤ 2. In particular, *ℋ* is not a 0-dimensional sheaf. Moreover, *ℋ* is a quotient sheaf of *𝒪*_*T*_1__ with constant term 1 and so we have *ℋ*≅*𝒪*_*T*_3__ with *T*_3_ ⊂ *T*_1_ and *T*_3_ ∈ |*𝒪*_*Q*_(1,1)|. For example, if *T*_3_ = *T*_1_, then we have *𝒦*′ = 0 and it contradicts the nontriviality of the extension *ℱ*. Thus, *ℱ* also admits the sequence

(61) $$\begin{array}{c} 0⟶𝒦⟶ℱ⟶{𝒪}_{T_{3}}⟶0, \end{array}$$

where *χ*_*𝒦*_(*x*, *y*) = *x* + *y* + 1. Since *𝒦*′ is a subsheaf of *𝒪*_*T*_1__ with *χ*_*𝒦*′_(*x*, *y*) = *x*, we have *𝒦*′≅*𝒪*_*T*_4__(−1,0), where *T*_4_ is a subcurve of *T*_1_ such that *T*_1_ = *T*_3_ + *T*_4_. Thus, *𝒦* is an extension of *𝒪*_*T*_2__ by *𝒪*_*T*_4__(−1,0). It is nontrivial, otherwise we would have *𝒪*_*T*_2__ as a direct factor of *ℱ*. Since there exists such a unique extension *𝒪*_*T*_2_+*T*_4__, *ℱ* admits an extension of *𝒪*_*T*_3__ by *𝒪*_*T*_2_+*T*_4__:

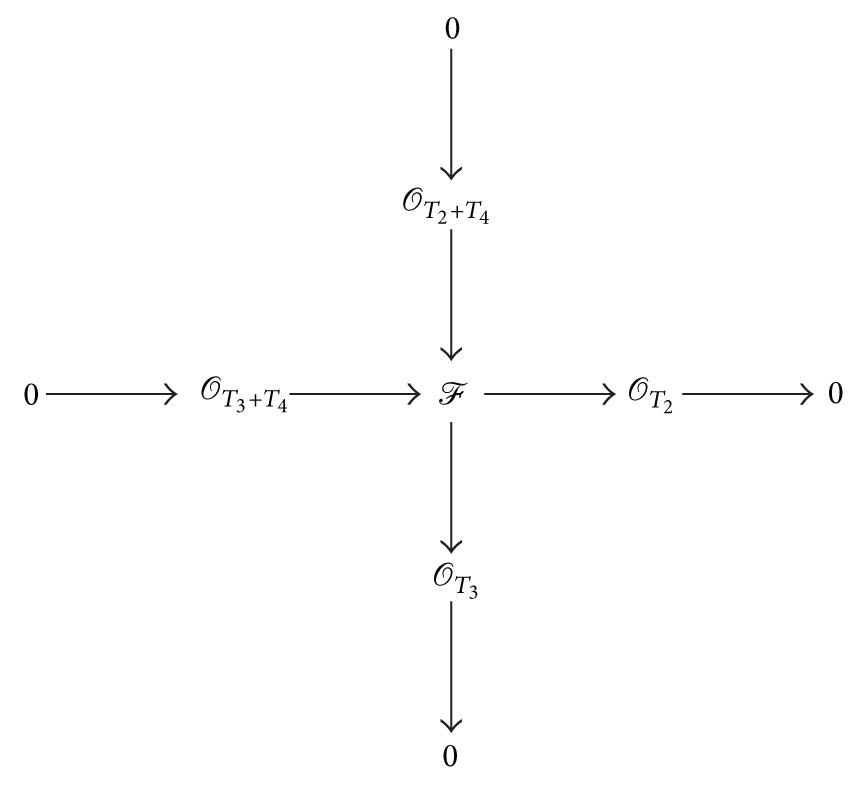
(62)
It implies that *ℱ* is of type (B).

**Lemma 26**

Let *ℱ* be a line bundle on a reduced curve *C* ∈ |*𝒪*_*Q*_(2,2)| with degree 2.

1. *ℱ* is semistable if and only if one has:
  - deg⁡(*ℱ*|_*T*_) ≥ 1 for all subcurves *T* of *C* in |*𝒪*_*Q*_(*a*, *b*)| with (0,0)⪇(*a*, *b*)⪇(1,1),
  - deg⁡(*ℱ*|_*A*_) ≥ 0 for each smooth subcurve *A* of *C* in |*𝒪*_*Q*_(*a*, *b*)| with (1,1)≤(*a*, *b*)⪇(2,2).
2. *ℱ* is stable if and only if deg⁡(*ℱ*|_*T*_) ≥ 1 for all subcurves *T* of *C* in |*𝒪*_*Q*_(*u*, *v*)| with (0,0)⪇(*u*, *v*)⪇(2,2).

**Proof**

In both parts, the “only if” part is obvious. Assume that *ℱ* is not stable (resp., semistable) and take a proper subsheaf *ℋ* of *ℱ* with *p*(*ℋ*) ≥ *p*(*ℱ*) (resp. *p*(*ℋ*) > *p*(*ℱ*)). Taking a saturation of *ℋ* in *ℱ*, we may assume that *𝒢* : = *ℱ*/*ℋ* is a pure sheaf. Call *A* the scheme support of *ℋ* and *T* the scheme support of *𝒢*. The definition of scheme support of a purely 1-dimensional sheaf gives *A* + *T* = *C* as effective divisors. Thus *T* has one of the types in the assertion. Since *C* is reduced and *ℱ* is a line bundle on *C*, the support of *𝒢* must be a proper subcurve *T* of *C*. If *T* does not have a type of *𝒪*_*Q*_(1,2) or *𝒪*_*Q*_(2,1), then we are done. But the case of *T* having such types is excluded using the argument in the proof of Lemma 18.

**Lemma 27**

One has *𝔅*∩*ℭ* ≠ *∅*.

**Proof**

Let us set *B* = *B*′ + *T*_2_ with *B*′ ∈ |*𝒪*_*Q*_(0,1)| and *T*_2_ ∈ |*𝒪*_*Q*_(1,0)|, and set *A* ∈ |*𝒪*_*Q*_(1,1)| to be smooth. For any extension *ℱ* ∈ *𝔅* of *𝒪*_*B*_ by *O*_*A*_, for example, *ℱ* = *𝒪*_*A*_ ⊕ *𝒪*_*B*_, let *ℋ* be the kernel of the composition *ℱ* → *𝒪*_*B*_→*𝒪*_*T*_2__ and then *ℋ* is a pure sheaf with *T*_1_ : = *A* + *B*′ as its scheme support and has Hilbert bipolynomial *χ*_*ℋ*_ = *χ*_*𝒪*_*T*_1___. Note that it has *𝒪*_*A*_ as its subsheaf.

To prove *ℋ*≅*𝒪*_*T*_1__, it is sufficient to prove that *ℋ* is semistable. Suppose *ℋ* is not semistable and take a proper saturated stable subsheaf *𝒢* ⊂ *ℋ* with *χ*_*𝒢*_ = *ax* + *by* + *c*. Its scheme support is contained in *T*_1_ and it is of type (*b*, *a*). Without loss of generality, let us assume that *a* ≤ *b*. First, assume (*a*, *b*) = (1,2). In this case, we would have *c* ≥ 2 because *p*(*𝒢*) > *p*(*ℋ*) and so we have *h* ^0^(*ℋ*) ≥ 2, contradicting the fact that *h* ^0^(*ℱ*) = 2 and that *ℱ* is globally generated. Assume *a* = *b* = 1. The map *𝒢* → *ℋ* on *A*∖*T*_2_ must be just the inclusion *𝒪*_*A*_ → *ℋ*, because *ℋ*|_*A*∖*T*_2__ is a line bundle. Thus either we have *𝒢* = *𝒪*_*A*_ or *𝒪*_*A*_ is not saturated in *ℋ*. Hence the saturation *𝒜* of *𝒪*_*A*_ in *ℱ* has slope greater than 1/2, contradicting the semistability of *ℱ*. Now assume *a* = 0 and *b* = 1, that is, *C*_*𝒢*_ = *B*′. Since *B*′ is smooth, *𝒢* is a line bundle on *B*′. If its degree *d* is at least 1, then *𝒢* contradicts the semistability of *ℱ*. If *d* ≤ 0, then we have *p*(*𝒢*) < *p*(*ℋ*), a contradiction. Hence *ℱ* is also contained in *ℭ*.

**Lemma 28**

For *T*_1_ ∈ |*𝒪*_*Q*_(1,2)| and *T*_2_ ∈ |*𝒪*_*Q*_(1,0)|, one has

(63) $$\begin{array}{c} \dim\;\text{Ex}{\text{t}}^{\text{1}}\left({𝒪}_{T_{2}},{𝒪}_{T_{1}}\right)=2. \end{array}$$

**Proof**

Applying the functor Hom(*𝒪*_*T*_2__, −) to the sequence of *T*_1_, we obtain

(64) 0⟶Ext⁡1(𝒪T2,𝒪Q)⟶Ext⁡1(𝒪T2,𝒪T1)⟶Ext⁡1(𝒪T2,𝒪Q(−1,−2))⟶0,

since we have

(65) Ext1(𝒪T2,𝒪Q(−1,−2))≅H1(𝒪T2(−1,0))∨≅H1(𝒪T2)∨≅H0(𝒪T2(−2))=0

and similarly Ext^2^(*𝒪*_*T*_2__, *𝒪*_*Q*_) = 0. Note also that Ext^1^(*𝒪*_*T*_2__, *𝒪*_*Q*_)≅*H* ^0^(*𝒪*_*T*_2__) and Ext^2^(*𝒪*_*T*_2__, *𝒪*_*Q*_(−1, −2))≅*H* ^0^(*𝒪*_*T*_2__)^∨^. Thus we have the assertion.

**Remark 29**

When *T*_1_ and *T*_2_ meet transversally at two points, say *P*_1_ and *P*_2_, then Ext^1^(*𝒪*_*T*_2__, *𝒪*_*T*_1__) is the global sheaf of a sheaf with support on *P*_1_ and *P*_2_ with one copy of *ℂ* on each point *P*_1_, *P*_2_,

(66) $$\begin{array}{c} \text{Ex}{\text{t}}^{1}\left({𝒪}_{T_{2}},{𝒪}_{T_{1}}\right)≅{\mathbb{C}}_{P_{1}}⊕{\mathbb{C}}_{P_{2}} \end{array}$$

for the following reason.

Let *R* be a regular local ring of dimension 2 and take *x*, *y* generators of its maximal ideal. All Ext^*i*^ groups are with respect to *R*. Since *R*/(*y*) is Gorenstein, so the duality gives Ext_*R*_^1^(*R*/(*y*), *R*)≅*R*/(*y*) and Ext_*R*_^*i*^(*R*/(*y*), *R*) = 0 for all *i* ≠ 1. From the exact sequence

(67) $$\begin{array}{c} 0⟶R\overset{u}{⟶}R⟶\frac{R}{\left(x\right)}⟶0, \end{array}$$

in which *u* is the multiplication by *x*, we get that Ext_*R*_^1^(*R*/(*y*), *R*/(*x*)) is the cokernel of the multiplication by *x* in *R*/(*y*) → R/(*y*); that is, we have Ext_*R*_^1^(*R*/(*y*), *R*/(*x*)) = *ℂ*. The same is true for extensions of *𝒪*_*B*_ by *𝒪*_*A*_ when *A* and *B* are transversal.

**Lemma 30**

Let *ℱ* be a sheaf of type (*A*) with no 0-dimensional torsion. Then *ℱ* is semistable unless it admits the sequence

(68) $$\begin{array}{c} 0⟶{𝒪}_{T_{2}}⟶ℱ⟶{𝒪}_{T_{1}}⟶0, \end{array}$$

where *T*_1_ ∈ |*𝒪*_*Q*_(*a*, *b*)| and *T*_2_ ∈ |*𝒪*_*Q*_(2 − *a*, 2 − *b*)| with (*a*, *b*)∈{(1,2), (2,1)}.

**Proof**

Let *𝒦* be a subsheaf *ℱ* with maximal *p*-slope *p*(*𝒦*) > 1/2 and so the quotient sheaf *ℋ* : = *ℱ*/*𝒦* has no 0-dimensional torsion. Let us set *χ*_*𝒦*_(*x*, *y*) = *m*′*x* + *n*′*y* + *t*′ with *t*′ ≥ 1 and (0,0)⪇(*m*′, *n*′). If the composite *s* : *𝒦*↪*ℱ*²↠*η* is a zero map, then *𝒦* is a subsheaf destabilizing *𝒪*_*C*_, a contradiction. If *s* is not surjective, for instance, *Im*⁡(*s*) = *𝒪*_*P*_⊊*η*, then *ker*⁡(*s*) is a subsheaf of *𝒪*_*C*_ with Hilbert bipolynomial *m*′*x* + *n*′*y* + *t*′ − 1. Thus we have *t*′ = 1 and the quotient *ℋ*′ : = *𝒪*_*C*_/*𝒦*′ has Hilbert bipolynomial with zero constant term. Since *ℋ*′ has no 0-dimensional torsion, we have *ℋ*′≅*𝒪*_*D*_ for a curve *D* contained in *C*. But the Hilbert bipolynomial of *𝒪*_*D*_ has nonzero constant term, a contradiction. Thus the map *s* is surjective. Following the same argument before, we obtain that *t*′ = 1 and *m*′ + *n*′ ≤ 1. Without loss of generality, let us assume that (*m*′, *n*′) = (0,1). Then we have *χ*_*ℋ*_(*x*, *y*) = 2*x* + *y* + 1 and thus we have *ℋ*≅*𝒪*_*T*_1__, where *T*_1_ is a curve contained in *C*_*ℱ*_ and *T*_1_ ∈ |*𝒪*_*Q*_(1,2)|. Since *𝒦* is a subsheaf of *ℱ* with *χ*_*𝒦*_(*x*, *y*) = *y* + 1, we have *𝒦*≅*𝒪*_*T*_2__ since *𝒦* has no 0-dimensional torsion. Thus *ℱ* fits into the sequence (68).

**Remark**

Applying the functor Hom(*η*, −) to the sequence of *C* ∈ |*𝒪*_*Q*_(2,2)|, we obtain

(69) 0⟶Ext⁡1(η,𝒪C)⟶Ext⁡2(η,𝒪Q(−2,−2))⟶fExt⁡2(η,𝒪Q).

Since the map *f* is the dual of the map Hom(*𝒪*_*Q*_(2,2), *η*) → Hom(*𝒪*_*Q*_, *η*) given by the multiplication by the defining equation of *C*, the map *f* is a zero map and thus we have Ext^1^(*η*, *𝒪*_*C*_)≅*H* ^0^(*η*)^∨^. In particular its dimension is 2.

**Lemma 32**

Let *ℱ* be a sheaf of type (*B*) fitting into an exact sequence

(70) $$\begin{array}{c} 0⟶{𝒪}_{T_{1}}⟶ℱ⟶{𝒪}_{T_{2}}⟶0 \end{array}$$

with *T*_1_, *T*_2_ ∈ |*𝒪*_*Q*_(1,1)|. Then *ℱ* is of type (A) if and only if *T*_1_ and *T*_2_ have no common components; that is, *C*_*ℱ*_ has no multiple component.

**Proof**

If *T*_1_ and *T*_2_ have a common component, say *T*, then *ℱ* has rank 2 at the general point of *T* and thus *ℱ* is not of type (A).

Conversely, assume that *T*_1_∩*T*_2_ is finite. Since we have *h* ^1^(*𝒪*_*T*_1__) = 0, the sequence (70) implies that *h* ^0^(*ℱ*) = 2 and *ℱ* is globally generated. Let *σ* be a general section of *ℱ* and then it does not vanish at the general point of any of the components of *C*_*ℱ*_. Since *C*_*ℱ*_ is reduced, *σ* induces an injective map *𝒪*_*C*_*ℱ*__↪*ℱ* and thus *ℱ* has type (A).

**Lemma 33**

Let *ℱ* be a sheaf of type (*C*).

1. If *T*_2_ is not a component of *T*_1_, then *ℱ* is of type (A).
2. If *T*_2_ is a double component of *C*_*ℱ*_, that is, *T*_2_ ⊂ *T*_1_, then it is not of type (A).

**Proof**

Let us assume that *T*_2_ ∈ |*𝒪*_*Q*_(1,0)|.

1. Since *T*_2_ is not a component of *T*_1_, *ℱ* is a line bundle on *C* = *C*_*ℱ*_ outside finitely many points of *C*. Moreover, it is not an *𝒪*_*T*_1__-sheaf. Note that *ℱ* is globally generated since *𝒪*_*T*_1__ and *𝒪*_*T*_2__ are globally generated with *h* ^1^(*𝒪*_*T*_1__) = 0. Thus, a general section of *ℱ* does not vanish at a general point of *T*_2_ and so it does not induce an injection *𝒪*_*T*_1__↪*ℱ*. Hence, *ℱ* fits into some sequence (46).
2. Let us set *C* = 2*T*_2_ + *T*_3_ and *T*_1_ : = *T*_2_ + *T*_3_, where *T*_3_ ∈ |*𝒪*_*Q*_(0,2)|. Let Γ be the projectivisation of *Ext*⁡^1^(*𝒪*_*T*_2__, *𝒪*_*T*_1__) and in particular we have Γ≅*ℙ* ^1^ by Lemma 28. We also know from Lemma 25 that any *e* ∈ Γ gives a semistable sheaf. Such a sheaf has rank 2 at the points of *T*_2_∖(*T*_2_∩*T*_3_) and, in particular, it is not a line bundle over its support at a general point of *T*_2_. Thus, it never fits into an exact sequence (46). Otherwise it would be locally free of rank 1 at each point of the support of *T*_3_ but not in *T*_2_.

In general, the question whether the variety *𝒫ic*_(*m*,*n*)_^*d*^ is rational is difficult as in the projective plane. We observed that **M**_1_ is rational and so is *𝒫ic*_(2,2)_^1^. Below we give a partial answer to this question in the case of *𝒫ic*_(2,2)_^2^.

**Theorem 34**

**M**_2_ is unirational with degree 4.

**Proof**

Let us fix a smooth curve *C* of bidegree (2,2) in *Q* and a point *P* ∈ *C* to consider a sheaf *𝒪*_*C*_(*P*) ∈ **M**_1_. If *𝕋*_*P*_ is the tangent plane of *Q* at *P*, then we have *𝕋*_*P*_∩*C* = {2*P*, *Q*_1_, *Q*_2_} for some points *Q*_1_, *Q*_2_ on *Q* since deg⁡(*C*) = 4. It defines a rational map

(71) $$\begin{array}{c} \Phi :M_{1}⇢M_{2}, \end{array}$$

sending *𝒪*_*C*_(*P*) to *𝒪*_*C*_(*Q*_1_ + *Q*_2_). Note that *𝒪*_*C*_(*Q*_1_ + *Q*_2_) = *𝒪*_*C*_(1,1)(−2*P*). We claim that the map Φ is generically 4 to 1 and so the assertion follows.

Let *U* (resp., *V*) be the dense open subset of **M**_1_ (resp., *𝒜* ⊂ **M**_2_) formed by the sheaves *ℱ* such that *C*_*ℱ*_ is smooth. Each element of *U* (resp., *V*) is uniquely determined by a smooth *C* ∈ |*𝒪*_*Q*_(2,2)| and a degree one (resp., degree two) line bundle on *C*. By Riemann-Roch, each degree one line bundle on *C* is associated with a unique *P* ∈ *C*. Then the map Φ sends *𝒪*_*C*_(*P*) to *ℛ* : = *𝒪*_*C*_(1,1)(−2*P*). Fix any degree two line bundle *ℳ* on *C*. Since we are in characteristic zero, there are exactly four line bundles *𝒜* on *C* such that *𝒜* ^⊗2^≅*𝒪*_*C*_. Hence, for each *ℛ* ∈ Pic^2^(*C*) there are exactly 4 points *P* ∈ *C* such that *ℛ*≅*𝒪*_*C*_(1,1)(−2*P*). Hence, Φ is dominant and the preimage of each element of *V* has cardinality 4.

We did not succeed in getting any smaller degree of unirationality of **M**_2_ as of now, and we left the rationality question as a conjecture.

**Conjecture 35**

**M**_2_ is rational.
